# An 18-Year-Old Patient with Type 1 Diabetes Undergoing Surgery

**DOI:** 10.1371/journal.pmed.0020140

**Published:** 2005-05-31

**Authors:** Massimo Pietropaolo

## Abstract

A young patient with type 1 diabetes needs an elective operation under general anesthesia. How will you manage his diabetes before, during, and after the surgery?

## DESCRIPTION of CASE

An 18-year-old Caucasian male with type 1 diabetes presented to the emergency department complaining of severe left knee pain and swelling after sustaining a knee injury that occurred during a high school football match. Joint effusions were visible and palpable above the left knee, and there was significant loss of smooth motion of the knee, passively performed. Plain X rays showed no signs of fractures. The patient had had type 1 diabetes for six years, and his insulin regimen consisted of insulin glargine, 35 units at 8:00 p.m., and insulin lispro, 23 units at 8:00 a.m. and 16 units at 8:00 p.m. The patient had no apparent complications related to type 1 diabetes.

On examination he was alert, his pulse was 76 bpm regular, and his blood pressure was 118/66 mm Hg. Recently, the patient had had frequent episodes of both hyperglycemia and hypoglycemia. However, he had never developed diabetic ketoacidosis (DKA). His recent HbA1c was 9.5%, demonstrating inadequate glycemic control.

The patient was referred to an orthopedic surgeon, and arthroscopy was scheduled a few days later. A complex tear of the medial meniscus extending to the articular surfaces was diagnosed. Partial meniscectomy was recommended. (This procedure usually takes about one hour—nonetheless, the preoperative preparation for general anesthesia and the postoperative recovery may add several hours to this time.)

### When Would You Have This Patient Report to the Hospital? The Day before Surgery or the Morning of Surgery?

This patient should be hospitalized no later than the evening before surgery, given his history of frequent episodes of hypo- and hyperglycemia and his poor glycemic control. This should allow for final optimization of glucose control before surgery. Ideally, frequent contact with the patient and adjustment of the insulin regimen prior to surgery should lead to an excellent glycemic control. This 18-year-old patient on glargine might benefit from additional doses of lispro given before lunch and dinner (rather than at 8 p.m., unless this is before dinner).

A patient with poorly controlled diabetes should not undergo elective surgery until glycemic levels are reasonably controlled. For example, bringing the patient into the hospital with blood glucose levels of 450 mg/dl will likely result in a canceled surgery. If a patient is scheduled for day of admission surgery, one way to avoid having to send the patient out without being able to do the procedure is to perform a finger stick blood sugar on arrival so one can cancel before admission.

Patients undergoing minor surgical procedures (e.g., arthroscopy) may be brought to the hospital on the morning of surgery. As the patient is usually NPO (i.e., has been given orders to fast before the surgery), a lower dose of the intermediate- or long-acting insulin is administered, and the regular insulin is withheld (Box 1). Insulin therapy should never be withheld in a patient with type 1 diabetes as this can result in DKA.

**Box 1. Management of Type 1 Diabetes during Minor Surgery Procedures**

Day of procedure if the patient is NPO:

- Withhold morning dose of insulin.
- Measure capillary glucose levels prior to procedure every 2–4 hours.
- Administer short-acting or fasting insulin subcutaneously every 2–4 hours as indicated in Table 1.
- Administer the usual afternoon dose of insulin.

Day of procedure if breakfast is allowed:

- Administer morning dose of insulin.
- Measure glycemia before and after procedure.
- Give supplemental four units of short- or fast-acting insulin subcutaneously if glycemic levels are >250 mg/dl.
- Give usual afternoon insulin dose.

Modified from [6].

### How Should His Insulin Be Managed before Surgery?

The degree of metabolic control should be carefully evaluated before surgery; the goal is to improve the patient's blood glucose readings on an outpatient basis before undergoing surgery. If hyperglycemia has been present for a prolonged period of time before surgery, this could result in dehydration, which is commonly associated with electrolytic abnormalities such as sodium and potassium loss and possibly intravascular volume depletion. Prolonged hyperglycemia will delay healing and increase the risk of ischemia. A number of observations have indicated that hyperglycemia impairs collagen formation and causes a decrease in the tensile strength of surgical wounds [1,2]. Simply avoiding hyperglycemia can prevent these consequences. The reduction of glucose levels to below 200 mg/dl has been shown to improve granulocyte adherence and granulocytosis, both key components of the innate immunity and the defense against bacterial infections. Studies in both humans and animals suggest that high glucose levels might exacerbate ischemic brain damage [3].

Admission to the hospital is recommended for all patients with type 1 diabetes, and a stabilization period of 12–16 hours is also recommended for urgent procedures if severe hyperglycemia is present [4,5]. However, the widespread use of home blood glucose monitoring makes the improvement of glycemic control possible prior to admission. Traditionally, long-acting (e.g. ultralente) insulin is discontinued 2–3 days before surgery, and the patient is stabilized on a regimen of intermediate-acting (neutral protamine hagedorn [NPH] or lente) and short-acting (regular or humalog) insulin twice a day, or regular insulin before meals and intermediate-acting insulin at bedtime. However, if the glycemic control is good and the patient is being treated with glargine, it is acceptable to continue the regimen until the day of surgery [3]. Alternatively, 1/2–2/3 of the usual insulin regimen is given on the day of the procedure [3]. On the morning of surgery patients with type 1 diabetes should receive the insulin regimen that is used intraoperatively (Box 2) [6].

**Box 2. IV Insulin-Glucose Infusion for Surgery**

Preoperative Days

Attempt to obtain reasonable glycemic control. The goal is to achieve preprandial glycemic levels between 70 and 150 mg/dl (3.9–8.3 mmol/l).

Operative Day

- Four to eight hours before surgery, keep patient NPO, discontinue SC insulin, and insert IV infusion line.
- Measure capillary glucose levels at one-hour intervals.
- Infuse D5W intravenously via an IV regulator pump.
- If renal function and serum potassium concentration are normal, add 20 mEq KCl/l.
- Based on hourly blood glucose determination, adjust each infusion as indicated in Table 2.

Modified from [6].

Evaluation of metabolic homeostasis, lipid profile, and kidney and myocardial function must be completed before surgery. The presence of diabetic autonomic neuropathy should also be assessed prior to surgery because this condition predisposes to perioperative hypotension. In such patients meticulous monitoring of blood pressure and volume status is essential during the perioperative period [7].

The common agreement is that 4–8 hours before surgery, the patient should be kept NPO, subcutaneous (SC) insulin should be discontinued, and an intravenous (IV) infusion line should be inserted. It should be emphasized that if the elective surgical procedure can be scheduled for early morning hours and the procedure lasts at least four hours, this limits the NPO to about 4–6 hours and allows administration of usual or 1/2–2/3 usual insulin and use of a glucose infusion to replace breakfast and supplemental insulin either subcutaneously or intravenously. If both glucose and insulin are infused, it should be pointed out that these must be two separately controlled infusions, so glucose and insulin infusion can be varied independently.

### How Should His Insulin Be Managed during Surgery?

Major surgery and general anesthesia can cause severe metabolic abnormalities in patients with type 1 diabetes. Given the limitations of SC insulin therapy, such as unpredictable absorption and variable plasma insulin levels, constant infusion of insulin is recommended [3]. Anesthesia induces complex neuroendocrine stress responses and activates the sympathetic nervous system. The abnormal release of growth hormone, cortisol, and epinephrine leads to impaired insulin secretion, and causes insulin resistance and hyperglycemia due to increased glycogenolysis, gluconeogenesis, and decreased glucose disposal. Many other conditions may cause severe insulin resistance in a patient with diabetes (Box 3).

**Box 3. Illnesses and Conditions Frequently Associated with Increased Insulin Requirement**

- Recent DKA
- Poor glycemic control
- Sepsis
- Steroid therapy
- Liver disease
- Obesity

For patients scheduled for elective surgery, IV insulin and glucose infusion are usually started several hours preoperatively, and glucose levels should be maintained between 100 and 125 mg/dl. Slightly higher targets (100–150 mg/dl) have been recommended by some diabetologists to minimize the risk of hypoglycemia. The maintenance of glucose levels below 200 mg/dl has been shown to prevent bacterial infections and ischemic brain damage [3,8]. Suggested guidelines for management of patients with type 1 diabetes by use of insulin are outlined in Box 2. Since IV regular insulin has a short half-life (ten minutes), hypoglycemia is of little concern, as the infusion can be decreased and the IV glucose rate increased. The infusion rate can be adjusted by a floor nurse before surgery and by the anesthesiologist intraoperatively. An insulin infusion algorithm is constructed to allow easy titration of the insulin dose, and IV glucose must also be infused to obtain glucose levels within target (Box 2). This type of algorithm is effective in the majority of patients; however, it is based on the “average” patient and might require individualization. If the patient has a coexisting condition associated with increased insulin requirement, IV insulin doses need to be increased. Glycemic levels must be monitored at hourly intervals to keep glucose levels between 100 and 125 mg/dl.

Patients with type 1 diabetes who are treated with continuous SC insulin infusion by an insulin pump should be easily converted to IV regular insulin infusion just before surgery. Continuous SC insulin infusion is an acceptable regimen for surgical procedures requiring local anesthesia.

### How Should Fluids and Electrolytes Be Managed in This Patient?

An adult without diabetes requires a minimum of 100 to 125 grams (400 to 500 calories) of glucose per day to prevent protein catabolism and the development of ketosis. Hence, this patient with types 1 diabetes should be treated with 5–10 grams of glucose per hour (1.2 to 2.4 mg/kg/min in a 70-kilogram subject) to provide sufficient basal energy requirement and prevent hypoglycemia during surgery. The dextrose concentration of the IV solution (Box 2) is adjusted based on the expected length of surgery. Thus, in this case 5% of dextrose in water (D5W) can be administered intravenously via infusion pump. For longer surgical procedures (intraabdominal or intrathoracic surgery), 10% of dextrose should be used to avoid excessive fluid administration. A 20% or 50% dextrose solution can be infused through a central venous catheter if fluid restriction is critical. If additional fluids are required, for instance, to replace unexpected intraoperative blood losses, non-glucose-containing solutions should be administered.

As a general rule, normal serum potassium levels do not necessarily imply that the total body potassium content is normal, as only 2% of total body potassium stores are extracellular [9]. In patients with diabetes, the metabolic homeostasis can rapidly be altered and many factors may influence serum potassium levels and total body potassium stores. These factors are (a) insulin, which increases potassium uptake by cells; (b) acidemia, which causes hyperkalemia as a result of the exchange of intracellular potassium for hydrogen ions; and (c) hyperosmolarity, which causes a rearrangement of potassium and fluid from intracellular to extracellular compartments. In patients with diabetes who have normal renal function and normal serum potassium concentration, 10 to 20 mEq of potassium should be added to each liter of dextrose-containing fluid. A higher dose is required in patients with hypokalemia. If serum potassium levels are greater than 5.5 mEq/l, potassium therapy should be withheld from the IV fluids and potassium serum levels should be monitored closely.

### How Would the Management of This Patient Be Different for Emergency Rather Than Elective Surgery?

It has been estimated that as many as 5% of all patients with diabetes require surgery at some point during their lives [3]. Many of these patients undergo emergency surgery as a result of lower extremity infections requiring incision and drainage or even lower limb amputations. More than 50% of lower limb amputations in the United States occur among people with diabetes. The majority of patients with diabetes admitted for emergency procedures have a poor glyco-metabolic control and some of them may have coexisting DKA. The first step in management is to assess glycemic, electrolyte, acid-base, and volume status. An IV saline infusion should be started while waiting for laboratory tests to correct possible volume loss. Insulin infusion should be started at an appropriate rate (Box 2), and frequently insulin requirement might increase during emergency surgery. It is crucial that volume losses and electrolyte abnormalities are corrected prior to surgery.

If DKA is diagnosed, immediate treatment is indicated, and, if possible, surgery should be delayed until the glyco-metabolic control is corrected and stabilized. If emergency surgery procedures cannot be delayed, DKA can be treated concurrently with surgery.

### How Should the Postoperative Glyco-Metabolic Management Be Handled in This Patient?

Both IV insulin and glucose (D5W, 0.45% normal saline) infusion should be continued until the glycol-metabolic control is stable and until 1–2 hours after the patient is able to resume oral feeding without difficulty. If postoperative nausea and vomiting are present, IV insulin and glucose infusion should not be discontinued. Furthermore, in patients with type 1 diabetes ketonuria could be an early sign of impending DKA, which could be triggered by starvation. Capillary glucose should be monitored every 1–2 hours at the bedside, and the variable insulin and glucose infusion should be adjusted to maintain blood glucose levels between 100 and 150 mg/dl (Box 4). Serum electrolytes should be measured immediately after surgery. Hypo- and hyperkalemia are fairly common in the postoperative period and should be corrected without delay. The presence of a widened anion gap suggests the possibility of DKA or lactic acidosis that might be caused by systemic infections or hypoperfusion.

**Box 4. Type 1 Diabetes Management after Surgery**

- Continue IV insulin and glucose (D_5_W) infusion until two hours after oral feeding is resumed.
- Measure capillary blood glucose before meals, at 10:00 p.m., and at 3:00 a.m.^a^
- Maintain blood glucose levels between 100 and 150 mg/dl (5.5–8.3 mmol/l).
- Provide three meals and three snacks (20–30 kcal/kg/day).
- Administer preprandial short- or fast-acting insulin subcutaneously according to the schedule in Table 3.
- For patients treated with continuous SC insulin infusion, resume basal rate and give bolus doses according to the carbohydrate counts.

^a^If hypoglycemia is present at 3:00 a.m., reduce the 10:00 p.m. insulin dose.

Modified from [6].

If the patient is stable and can tolerate oral feeding, the regular home dose of insulin may be administered 20–30 minutes before the meal and the insulin infusion stopped 15–20 minutes after the meal. Of note, insulin infusion should be discontinued only after the SC insulin regimen is started to avoid any gaps in plasma insulin levels that may lead to a loss of metabolic control.

Resuming a sliding scale SC insulin treatment postoperatively has been advocated to improve metabolic control in patients with type 1 diabetes. However, there are several drawbacks with this approach. First of all, this approach is based on a retrospective treatment for hyperglycemia, which reflects the degree of insulin sensitivity and glucose disposal rate in the preoperative period. The use of a SC insulin treatment postoperatively tends to create fluctuations in blood glucose levels that could be difficult to control. Moreover, there is a risk of exposing the patient to the risk of hypoglycemia if excessive doses of insulin are administered. More importantly, the use of a sliding scale treatment might predispose to DKA in insulin-deficient patients before the development of hyperglycemic levels.

### What Are the General Principles for Surgery in a Patient with Type 2 Diabetes?

Patients with type 2 diabetes require good blood glucose control prior to undergoing surgery. Although these patients seldom develop DKA, the same adverse effects of poor glycemic control may develop. For procedures that require general anesthesia, specific treatment, other than hourly glycemic monitoring, is not required for patients with type 2 diabetes whose diabetes is well controlled with diet (i.e., fasting blood glucose less than 125 mg/dl). However, insulin should still be administered as described in Box 2, as hyperglycemic responses may still occur intraoperatively in these patients. Targeted glycemic levels are identical to those for patients with type 1 diabetes. For elective procedures requiring general anesthesia, insulin infusion is usually required to control hyperglycemic levels that occur during surgery. Frequent intraoperative glucose monitoring is imperative in this setting to avoid complications resulting from a poor glycemic control. Patients with type 2 diabetes who require insulin and who are scheduled for surgery should be managed similarly to patients with type 1 diabetes.

As a general rule, SC insulin should not be used in patients with type 2 diabetes requiring surgery and general anesthesia, since insulin absorption from the SC tissue could be quite variable, particularly in obese individuals.

With regard to patients with type 2 diabetes who require general anesthesia and whose diabetes is well controlled with sulfonylureas, there is some degree of disagreement. Many diabetologists recommend withholding the sulfonylurea the morning of surgery for procedures requiring general or local anesthesia and using continuous insulin infusion.

Any patient treated with metformin should discontinue this medication at least 48 hours before surgery. The drug should be completely cleared after discontinuing metformin 48 hours preceding surgery. This is a prophylactic measure in an effort to lower the risk of lactic acidosis that could be secondary to complications of surgical procedures such as hypotension, myocardial infarction, or septic shock.

Treatment decisions for patients with type 2 diabetes receiving local anesthesia and undergoing minor surgical procedures are similar to those described above for patients with type 1 diabetes.

## DISCUSSION

Patients with type 1 diabetes undergoing elective or emergency surgical procedures have a higher degree of morbidity and mortality than those without diabetes, as a result of impaired glyco-metabolic homeostasis and electrolyte balance. The magnitude of the catabolic responses is not only related to the severity of surgical and postsurgical complications but also to the effects of inadequate perioperative management on metabolic control. Health-care providers should be very familiar with the perioperative management of type 1 diabetes; with individualized insulin and glucose variable infusions, young patients affected by type 1 diabetes can undergo surgery with a minimal risk.

The goal of glycemic management in these situations is to maintain normal glucose homeostasis and normal metabolism. As insulin resistance and gluconeogenesis increase during surgery-related stress and anesthesia, additional insulin will be needed to prevent excessive hepatic glucose release. An important issue is maintaining a physiological fluid and electrolyte balance.

**Key Learning Points**

- Patients with type 1 diabetes undergoing elective or emergency surgical procedures have a higher degree of morbidity and mortality than those without diabetes.
- Perioperative hyperglycemia is associated with an increased risk of infections, delayed wound healing, and an increased risk of ischemia.
- A patient with poorly controlled diabetes should not undergo elective surgery until glycemic levels are reasonably controlled.
- Patients undergoing minor surgical procedures, such as arthroscopy, may be brought to the hospital on the morning of surgery.
- Given the limitations of subcutaneous insulin therapy, such as unpredictable absorption and variable plasma insulin levels, constant infusion of insulin is recommended during surgery.

Perioperative hyperglycemia increases the risk of infections, delayed wound healing, and ischemia. Achieving preprandial glycemic levels between 70 and 150 mg/dl before surgery in the preoperative period and maintaining plasma glucose levels between 100 and 125 mg/dl during surgery and between 100 and 150 mg/dl after surgery using simple and safe algorithms (Boxes 1, 2, and 4) can significantly decrease the risk of these complications.

**Table 1:**
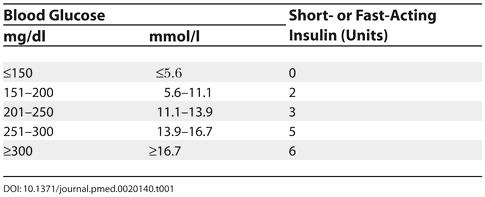
Administering Insulin during Minor Surgery Procedures

**Table 2:**
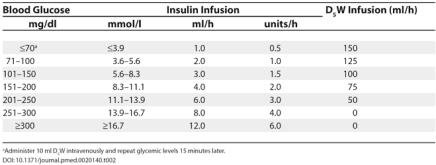
Infusing Insulin-Glucose during Surgery

^a^ Administer 10 ml D_5_W intravenously and repeat glycemic levels 15 minutes later.

**Table 3:**
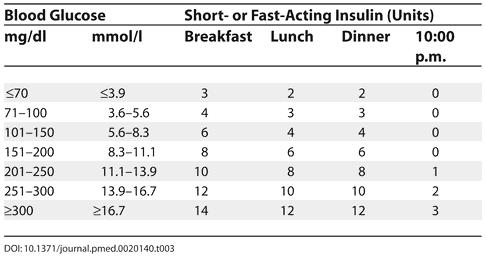
Administering Insulin after Surgery

## Abbreviations

D5W: 5% percent of dextrose in water
DKA: diabetic ketoacidosis
IV: intravenous
SC: subcutaneous

## References

[pmed-0020140-b1] Marhoffer W, Stein M, Maeser E, Federlin K (1992). Impairment of polymorphonuclear leukocyte function and metabolic control of diabetes. Diabetes Care.

[pmed-0020140-b2] Rosenberg CS (1990). Wound healing in the patient with diabetes mellitus. Nurs Clin North Am.

[pmed-0020140-b3] Clement S, Braithwaite SS, Magee MF, Ahmann A, Smith EP (2004). Management of diabetes and hyperglycemia in hospitals. Diabetes Care.

[pmed-0020140-b4] van den Berghe G, Wouters P, Weekers F, Verwaest C, Bruyninckx F (2001). Intensive insulin therapy in the critically ill patients. N Engl J Med.

[pmed-0020140-b5] Avilès-Santa L, Raskin P, Lebovitz HE (2004). Therapy for diabetes mellitus and its related disorders. Surgery and anesthesia.

[pmed-0020140-b6] Rosenstock J, Raskin P (1987). Surgery! Practical guidelines for diabetes management. Clin Diabetes.

[pmed-0020140-b7] Burgos LG, Ebert TJ, Asiddao C, Turner LA, Pattison CZ (1989). Increased intraoperative cardiovascular morbidity in diabetics with autonomic neuropathy. Anesthesiology.

[pmed-0020140-b8] Gill GV, Alberti KGMM, Alberti KGMM, Zimmet PZ, DeFronzo RA (1997). International textbook of diabetes mellitus. The care of the diabetic patient during surgery.

[pmed-0020140-b9] Levetan CS, Magee MF (2000). Hospital management of diabetes. Endocrinol Metab Clin North Am.

